# Four System Enablers of Large‐System Transformation in Health Care: A Mixed Methods Realist Evaluation

**DOI:** 10.1111/1468-0009.12684

**Published:** 2023-12-25

**Authors:** EMILIE FRANCIS‐AUTON, JANET C. LONG, MITCHELL SARKIES, NATALIE ROBERTS, JOHANNA WESTBROOK, JEAN‐FREDERIC LEVESQUE, DIANE E. WATSON, REBECCA HARDWICK, PETER HIBBERT, CHIARA POMARE, JEFFREY BRAITHWAITE

**Affiliations:** ^1^ Australian Institute of Health Innovation Macquarie University; ^2^ Centre for Primary Health Care and Equity University of New South Wales; ^3^ Bureau of Health Information St. Leonards; ^4^ Peninsula Medical School University of Plymouth

**Keywords:** realist evaluation, organizational change, innovation, complex adaptive systems, implementation science, health care reform, enablers of transformative change

## Abstract

**Policy Points**

- The implementation of large‐scale health care interventions relies on a shared vision, commitment to change, coordination across sites, and a spanning of siloed knowledge.
- Enablers of the system should include building an authorizing environment; providing relevant, meaningful, transparent, and timely data; designating and distributing leadership and decision making; and fostering the emergence of a learning culture.
- Attention to these four enablers can set up a positive feedback loop to foster positive change that can protect against the loss of key staff, the presence of lone disruptors, and the enervating effects of uncertainty.

**Context:**

Large‐scale transformative initiatives have the potential to improve the quality, efficiency, and safety of health care. However, change is expensive, complex, and difficult to implement and sustain. This paper advances system enablers, which will help to guide large‐scale transformation in health care systems.

**Methods:**

A realist study of the implementation of a value‐based health care program between 2017 and 2021 was undertaken in every public hospital (*n* = 221) in New South Wales (NSW), Australia. Four data sources were used to elucidate initial program theories beginning with a set of literature reviews, a program document review, and informal discussions with key stakeholders. Semistructured interviews were then conducted with 56 stakeholders to confirm, refute, or refine the theories. A retroductive analysis produced a series of context‐mechanism‐outcome (CMO) statements. Next, the CMOs were validated with three health care quality expert panels (*n* = 51). Synthesized data were interrogated to distill the overarching system enablers.

**Findings:**

Forty‐two CMO statements from the eight initial program theory areas were developed, refined, and validated. Four system enablers were identified: (1) build an authorizing environment; (2) provide relevant, authentic, timely, and meaningful data; (3) designate and distribute leadership and decision making; and (4) support the emergence of a learning culture. The system enablers provide a nuanced understanding of large‐system transformation that illustrates when, for whom, and in what circumstances large‐system transformation worked well or worked poorly.

**Conclusions:**

System enablers offer nuanced guidance for the implementation of large‐scale health care interventions. The four enablers may be portable to similar contexts and provide the empirical basis for an implementation model of large‐system value‐based health care initiatives. With concerted application, these findings can pave the way not just for a better understanding of greater or lesser success in intervening in health care settings but ultimately to contribute higher quality, higher value, and safer care.

Health systems globally are at a crossroads. quality and safety improvements are typically driven by local, small‐scale initiatives that provide on‐the‐ground solutions to specific clinical problems. This approach has broadly delivered improved care over time, especially in notable examples such as the application of medical emergency teams,^1^ surgical checklists,^2^ and hand hygiene campaigns.^3^ However, aging populations and higher rates of chronic conditions are raising new challenges for management. Care remains episodic in nature, and advances in precision medicine, artificial intelligence, and other new therapies and technologies are making their way into care in a generally haphazard manner. Leaders are increasingly turning their attention to large‐system transformation programs to realign processes of care delivery to address these needs.

Large‐scale transformational change programs are increasingly being introduced to address unprecedented pressure on services, low patient satisfaction, and poor outcomes, all of which collectively challenge both long‐term performance and sustainability of the system. By large‐scale transformation, we are referring to “coordinated, system‐wide change affecting multiple organizations and care providers, with the goal of significant improvements in the efficiency of health care delivery, the quality of patient care, and population‐level patient outcomes.”^4^ ^p422^ In contrast to clinician‐led, ground‐up, or localized change initiatives, large‐scale transformational change aims to affect more than local processes by standardizing practice across multiple sites, allowing better integration of services, and identifying and addressing the system's level dysfunction or barriers. A significant example of large‐scale transformation is the concept of value‐based health care, which is gaining international traction. Value‐based health care moves away from a volume‐orientated system to one that focuses on the value of outcomes for both patients and staff—at least, in theory.^5^

Implementation of the reforms needed to achieve system‐wide changes across multiple organizations and care providers is invariably expensive, complex, and difficult to implement. Prescriptive, top‐down approaches, which avoid duplication of effort, make accountabilities clear, and provide timely reporting of performance, have the aim of improving efficiency and patient care.^6^, ^7^ However, top‐down approaches that intend to simplify and improve health care systems may counterproductively create further complexities and barriers to care or system efficiency.^8^ For example, we found that situations in which audits are conducted by an external body without partnering with clinicians might not adequately capture local workflows.^9^ Clinicians may end up disengaged if the measures lack local meaning. Subsequently, mandatory audits conducted by an external body might be counterproductive, particularly for those clinicians whose reluctance has increased. Moreover, inadequate engagement and lack of local ownership are often identified as implementation barriers. A bottom‐up approach to implementation encourages and empowers local people to enact locally‐tailored solutions; however, change can be slow to happen. For successful implementation to occur, top‐down initiatives need to strike a balance between providing an intervention package that can be implemented by all, whilst preserving clinical autonomy at each site, and providing room for adaptation to contextual constraints. Top‐down initiatives are also challenging to evaluate.^10^

The methodology used to analyze top‐down and bottom‐up approaches to implementation can also present limitations. Policy implementation literature, which employs both positivist top‐down research and interpretive bottom‐up research, considers policies to be contestable and emergent in the complex process of interpretation and negotiation.^11^ Much of the implementation policy literature uses thick description, which has resulted in the implementation process being mapped or modeled rather than describing causal mechanisms to provide the foundation for universal theories.^12^ Peer‐reviewed empirical studies on large‐scale transformation in health service research are limited.^4^, ^10^, ^13^, ^14^, ^15^, ^16^, ^17^, ^18^ Those studies that have been done have made a significant contribution to the literature by conceptualizing the contexts and mechanisms for large‐scale transformation based on organizational case studies.^18^ We conducted a realist synthesis of large‐scale, multisite transformation programs drawn from peer‐reviewed and gray literature sources. We articulated 18 detailed contexts and mechanisms associated with increased or decreased implementer engagement with the initiative. One of the examples states, “When external support and/or endorsement of the proposed change is present, implementers may value the change more favorably or feel a greater tension for change resulting in increased engagement and commitment.”^18^

Successful large‐system transformation in health care systems has been characterized with reference to the properties of complex adaptive systems rather than conceived as top‐down change.^4^ Health care is made up of a daunting range of interdependent stakeholders who dynamically coadapt, learn, and self‐organize over time, leading to unpredictable and nonlinear patterns of behavior. For example, in certain circumstances, adherence to clinical practice guidelines can inadvertently increase disparities in health outcomes by discouraging personalized decision making.^19^

Although complex adaptive systems are intricate and unpredictable and change within them cannot be prespecified, they are amenable to guided transformation by applying “simple rules” that are sufficiently flexible to guide the fidelity of interventions but also to stimulate learning and adaptation. Best and colleagues^4^ identified five simple rules of large‐scale transformation that would likely increase the successful implementation of initiatives: (1) blend designated leadership with distributed leadership, (2) establish feedback loops, (3) attend to history, (4) engage physicians, and (5) include patients and families. Drawing on a realist review, this approach to the conceptualization of large‐scale transformation and the rules they produced are instructive and influential, providing a framework to understand the causal mechanisms that do (or do not) generate change. The five rules are, however, underdeveloped—a limitation that the authors acknowledge. There is scope, therefore, to advance our understanding of the universal properties that manifest during health system change efforts that will support successful implementation and scale up of value‐based health care. Creating this level of understanding demands multimethod, interdisciplinary research in real world settings to deeply apprehend the innovations and creativity of health care professionals to adapt to circumstances and evolve new and better ways of achieving quality.^20^ We use the term “system enabler” to refer to semipredictable patterns or behaviors or broad lessons of change for which interpretive flexibility would be needed in different contexts.

## Implementation Context

Leading Better Value Care (LBVC) is a flagship, value‐based health care program in NSW, Australia that aims to implement at scale evidence‐based models of care for patients with specific conditions and to assess the models’ impact over time. The core principle of value‐based health care in NSW expressly “considers what value means for patients, clinicians and the health system, and aims to provide health services that deliver value across four domains: improved health outcomes; improved experiences of receiving care; improved experiences of providing care; and better effectiveness and efficiency of care.”^5^ LBVC was collaboratively developed and administered by the NSW Ministry of Health (MoH) in partnership with adjunct policy bodies: the Agency for Clinical Innovation (ACI), Clinical Excellence Commission, and Cancer Institute NSW. In Australia, state and territory governments are responsible for health care planning with delivery by publicly funded hospitals. NSW Health provides universal access to health care services for Australia's most populous state (8 million people in 2021), which is operated by more than 130,000 staff spread across 221 public hospitals and facilities.^21^ The LBVC program was implemented by health districts and networks across more than 100 facilities in 2017, with eight initiatives in the first tranche.^5^ A further five initiatives were implemented in the second tranche from 2019. Seven of the first tranche initiatives were identified as having a high potential to reduce unnecessary hospitalization and were the primary focus of this realist study. These seven initiatives of interest are described in Table 1. Value‐based health care delivery models such as this, aim to address the unprecedented pressure on long‐term health system performance and sustainability and to respond to the changing needs and expectations of patients.^22^

**Table 1:** Description of the Seven LBVC Initiatives That Were the Focus of the Realist Study

Initiative	Patient Population	Aim
Osteoarthritis chronic care program	People with diagnosed osteoarthritis of the knee or hip	Improve daily function and delay and avoid or improve recovery from knee or hip joint replacement surgery
Osteoporosis refracture prevention	People < 50 y of age who present to hospital with osteoporotic fracture	Prevent refractures for people with osteoporosis by improving identification of conditions underlying minimal trauma fractures and streamlining case management processes
High‐risk foot service	People < 15 y of age with foot infections and/or ulcers of the foot or lower limb usually related to diabetes	Improve treatment and patient outcomes and reduce complications and associated hospitalizations for people with diabetes suffering from foot infections
Renal supportive care	People with chronic or end‐stage kidney disease, deciding whether to pursue renal replacement therapies, be conservatively managed or withdrawn from dialysis	Enhance patient (and carer) experience by supporting outpatient‐based symptom management and palliative care for people with chronic and end‐stage kidney disease
Inpatient management of diabetes mellitus	Acute admitted patients < 16 y of age with diabetes requiring subcutaneous insulin management	Reduce the length of hospital admission for people with diabetes requiring subcutaneous insulin by optimizing glucose management
Chronic heart failure	People < 18 y of age, admitted with symptoms suggestive of chronic heart failure	Reduce 28‐d readmission and 30‐d mortality by a focus on reducing unwarranted variation from best practice, enhance prevention, improve management and mitigation of risks
Chronic obstructive pulmonary disease	Acute admitted patients < 40 y of age with chronic obstructive pulmonary disease	Reduce 28‐d readmission and 30‐d mortality by a focus on reducing unwarranted clinical variation and optimization of lung function

We conducted a realist evaluation of the implementation of seven of these large‐system, value‐based health care initiatives in NSW, Australia.^23^ Realist research paradigms are theory‐led evaluations that offer an analytical tool to articulate the explicit role of context within the causal process, moving beyond a catalog of preconditions for implementation success.^24^ Realist studies assume that complex programs and interventions work differently under different circumstances and evaluate “what works, for whom, under what circumstance, and why.”^25^, ^26^ Realist evaluations tease out why an intervention that has been highly successful in one place is less successful (and even unsuccessful) in another place.

For example, clinical champions who are respected, credible, consistent, and clear can leverage their preexisting personal resources, network ties, as well as formal and informal authority to generate momentum for the initiative to become standard practice. Clinical champions, however, have less social influence in contexts where there is high staff turnover. This example is one of many generated from the study that provides some explanation of how and in what circumstances clinical champions enable change. This kind of study allows the identification of semipredictable patterns of change. A nuanced understanding of the contextual conditions for successful implementation may enable replication when implementing and scaling health care improvement programs.^27^, ^28^

Our aim was first to undertake a realist evaluation of the seven LBVC initiatives to understand how, why, and in what contexts the implementation of value‐based health care initiatives worked or did not work. Second, our aim was to distill system enablers from the data to guide implementers from leaders through to frontline staff.

## Methods

### Study Design and Rationale

**Figure 1:**
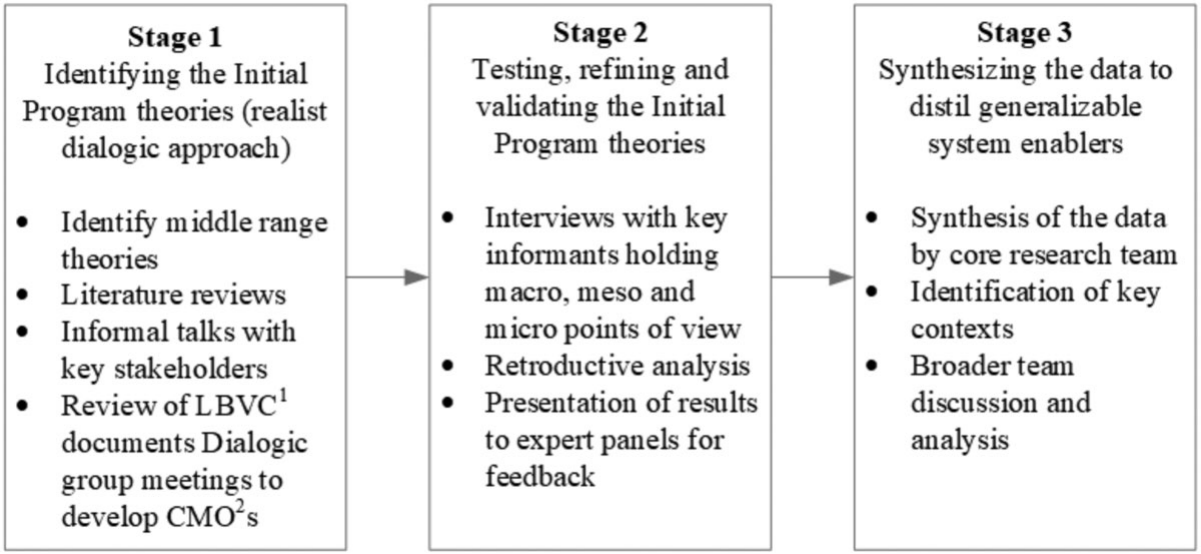
Three Stages of the Study CMO; context‐mechanism‐outcome; LBVC, Leading Better Value Care.

The study was conducted and reported according to our published protocol and followed the RAMESES II reporting standards for realist studies.^23^, ^29^ Data were collated from multiple sources (see Table 2) over different time periods to reinforce rigor and ensure that a diversity of perspectives were captured. Both qualitative and quantitative data were used to provide nuanced descriptions of the complex implementation process and substantiate observed patterns to ensure generalizability of explanations. The work was conducted in three stages: (1) identifying the initial program theories; (2) testing, refining, and validating the program theories; and (3) synthesizing the data to distill key, generalizable system enablers. Figure 1 provides an overview of the stages. Institutional Review Board (ethical) approval was obtained from Macquarie University (Ref 23816) and Hunter New England (Ref 2020/ETH02186) Human Research Ethics Committees.

**Table 2:** Data Sources for Realist Evaluation

Data Source	Number of Sources
Literature reviews^18^, ^31^	135 articles
Informal discussions with key program stakeholders	16 participants
Initiative document review	126 documents
Semistructured realist interviews with key informants	
NSW MoH (macrolevel)	6 participants
NSW ACI (mesolevel)	14 participants
Local hospitals (microlevel)	36 participants
Expert panels to validate findings and enablers	51 participants

ACI, Agency for Clinical Innovation; MoH, Ministry of Health; NSW, New South Wales.

#### Stage 1: Identifying the Initial Program Theories

We adopted a realist dialogic approach to develop our initial program theories, reported in detail elsewhere and summarized here.^30^ The process followed four phases: to (1) understand relevant theories, (2) review academic and gray literature, (3) conduct informal discussions with key stakeholders, and (4) undertake research‐group conversations. First, several sources of academic and gray literature were reviewed to identify hypotheses that could inform the development of an initial program theory for how and why the program was expected to work. This process included a systematic review of implementation determinants for hospital avoidance programs (*n* = 13 articles),^31^ a realist synthesis of implementation of large‐scale hospital improvement initiatives (*n* = 51 articles),^18^ targeted literature review of formal theoretical frameworks (*n* = 23 articles), and studies of large‐system transformation in health care (*n* = 48 articles). The systematic review and realist review formed an initial source of data to identify theory propositions that could then be situated within the LBVC program using other sources of data. As large‐scale transformation involves a suite of implementation strategies, each designed to address different concerns and involving different program theories, strategies were grouped into program theory areas (e.g., leadership, data monitoring, resource provision, audit and feedback). The development of the initial program theories started with the research team identifying and exploring relevant theories found through team knowledge or cited in realist or other studies. For instance, the leadership program theory drew on social capital theory,^32^, ^33^ social influence theory,^34^ and Friedson's^35^ theory of professions (see Francis‐Auton et al^30^ for a worked example).

Second, public documents (e.g., descriptions of the models of care, monitoring and evaluation plans, implementation plans, consultations, evidence reviews) pertaining to the LBVC program were reviewed to modify and situate these propositions within the LBVC program of interest. All documents were screened and included if they discussed concepts pertinent to the LBVC implementation or explicitly stated relevant program theories. These documents were analyzed retroductively alongside the identified initial program theories.

Third, when theory propositions were made specific to the LBVC program, informal discussions with key program stakeholders (∼ 16 stakeholders) were also used to map any differences between how the implementation was planned and how it was operationalized in practice. Finally, using David Bohm's concept of dialogue, we reached a common understanding of initial program theory through free‐flowing, cumulative, and genuine questioning and discussion within our core research team. We named this process a “dialogic realist approach.”^30^

#### Stage 2: Testing, Refining and Validating the Initial Program Theories

**Figure 2:**
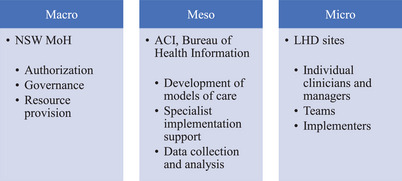
Conceptualization of Levels Within the Health System Involved in the LBVC Initiatives [Colour figure can be viewed at wileyonlinelibrary.com] ACI, Agency for Clinical Innovation; LHD, local health district; MoH, Ministry of Health; NSW, New South Wales.

We next tested and refined our initial program theories formally by conducting interviews with key stakeholders. We conceptualized these participants as holding macro, meso, or microlevel points of view in the NSW health system, depending on their role in the LBVC implementation (see Figure 2).

Semistructured realist interviews were conducted with 56 key stakeholders to build a nuanced understanding of how the LBVC program implementation occurred and why there was variation across different circumstances.^36^ We used maximum diversity sampling to obtain the greatest variety of organizational and individual perspectives and ensured we included as many of the 16 local health districts (LHDs) involved and an adequate spread of participants across the implementation strategies. Participants were invited to confirm, refute, or refine the initial program theories presented to them (see Supplementary File 1 for interview guides). The interviews were conducted via Zoom, audio recorded, and transcribed verbatim.

## Analysis

Retroductive analysis of the all data collected was undertaken using Nivo20, where the research team (M.S., E.F.A., C.P., N.R., J.C.L.) oscillated between inductive and deductive logic across many data sources and incorporated their insights.^37^ Transcripts or documents were first read in full to situate the data before researchers coded the transcripts line‐by‐line using a CMO configuration framework.^38^ A CMO configuration—(C) context or circumstances, (M) mechanisms or underlying social processes, and (O) outcomes or results—is a proposition‐building set of possible explanatory relationships between the components of realist studies.^38^, ^39^ Data were coded when an observable link between the contextual circumstances, mechanisms of change, and implementation outcomes for the implementation of the LBVC program were identified. The quotes were categorized as either supporting, refuting, or refining the CMO configuration framework, and the decision‐making process was recorded using memos. Approximately 20% of the coded transcripts or documents were checked by a second researcher to reinforce transparency and trustworthiness of the interpretations, and any disagreements between the researchers were resolved by discussion. Once coded, researchers engaged in group consensus, building meetings to finalize each program theory.^30^

### Development of System Enablers

Four presentations to key stakeholders from ACI (*n* = 11) and NSW MoH (*n* = 21) were held to validate and refine the final CMO statements and their interpretation. Participants were invited by members of the research team, targeting staff who held key positions at ACI or NSW MoH. These stakeholders were in senior positions and at the coalface of implementation at ACI and NSW MoH (e.g., clinical implementation and evaluation teams, divisional directors, and network managers from all the LBVC programs and members of the strategic reform branch of NSW MoH). E.F,A., J.C.L., and J.B. presented study findings via PowerPoint presentation, and participants offered further explanation or questions or highlighted key learnings that would impact their future practice.

This validation and refinement process led to some revisions of interpretation of the data. In addition, the work was presented to senior health service managers (*n* = 19) from across Australia. This focus group was held during the International Society for Quality in Health Care conference in Brisbane, Australia on October 14, 2022. The final expert panel focus group was advertised to Australian delegates holding senior health service management roles attending the conference (facilitated by conference organizers). Those who indicated interest were then formally invited and provided detailed information about the study. Comments and observations were used to further refine the interpretation of the CMO statements and to check their generalizability across different jurisdictions in Australia. The final refined set of CMO statements were read, synthesized, and discussed by the core research team (E.F.A., M.S., J.C.L., N.R., J.B.), and key themes, identified as system enablers, were extracted.

## Results

The final set of 42 CMO statements from eight initial program theory areas were developed and refined from all stages of data collection. The eight program theory areas that sought to explain how groups of implementation strategies were thought to work in different circumstances were: audit and feedback, business case for change, capability development, collaboration, data monitoring and evaluation, leadership, resource provision, and the “tight‐loose‐tight” approach (“tight” well‐defined targets and expected outcomes that are “loose” enough for local adaptation). The definitions of each program theory area and final CMO statements are shown in Supplementary File 2.

**Figure 3:**
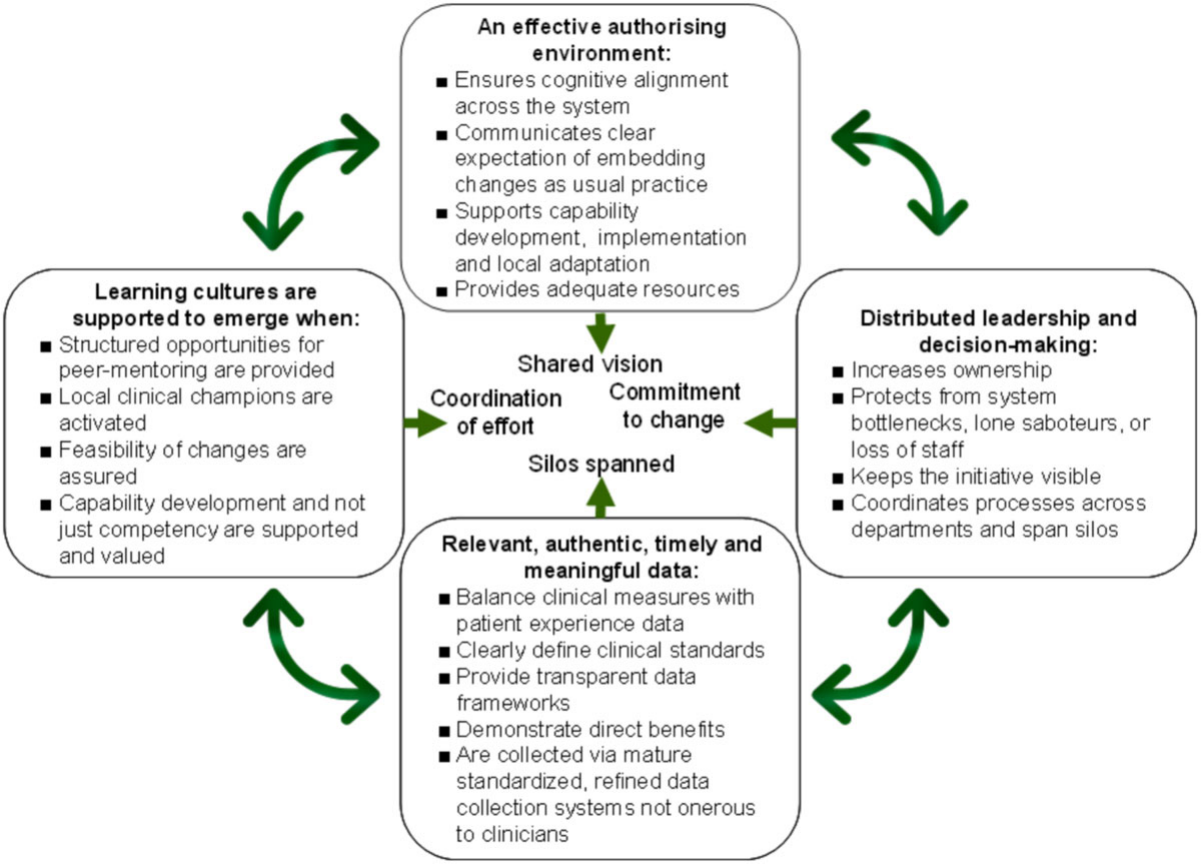
The Four System Enablers [Colour figure can be viewed at wileyonlinelibrary.com]

Four system enablers of large‐system transformation were distilled from these data: (1) build an authorizing environment; (2) provide relevant, authentic, timely, and meaningful data; (3) designate and distribute leadership and decision making; and (4) support the emergence of a learning culture (Figure 3). These system enablers are not standalone factors but are interdependent, complementary, and synergistic (i.e., they can set up useful feedback loops when working well). A summary of the enablers with supporting CMO statements is shown in Table 3.

**Table 3:** System Enablers and Related CMO Evidence

System Enabler	Supporting CMOs
Build an authorizing environment	
Cognitive alignment across nested levels of the system—statewide priority, health district/site priority, local team priority	Executive team and clinicians understand each other's responsibilities (M) and engagement with capability development increases (O) in situations where the need for training is valued and participation is actively facilitated (C).
	When local leaders engage key stakeholders through various forums, with reinforcement of the program by sponsors at a system level (C), this raises the profile and visibility of the initiative across departments (M), which, in turn, results in keeping the initiative on the agenda and ensuring consistency across the district (O).
	Overt or covert resistance to the initiative is cultivated at the clinician level (M) when there is a lack of transparency regarding decisions that affect them and at the executive‐level in situations with high leadership turnover, changing priorities, and uncertainty around the financial sustainability (C). Although the initiative can remain valued within different parts of the organization, operationalization becomes problematic under these circumstances (O).
Clear expectation of embedding change as usual practice	A case for change that is tailored to different audiences/specific priorities (C) enables genuine consensus building to take place between those advocating for the initiative and those responsible for delivering care (M), which builds a shared vision for the seamless integration into existing organizational structures, performance indicators, and activities (O).
	If there is uncertainty in the program's longevity or uncertainty in funding decisions (C), sustainability of the initiative is questioned (M). This leads to resources being spent unsuitably or absorbed into the organization's bottom line, preventing coordination and continuity of the initiative (O).
Capability development supported	Capability development activities and tools that address immediate clinical needs (C) open people's eyes to delivering care differently (M) and cultivating the knowledge, skill, and confidence needed to deliver the evidence‐based model of care (O).
Implementation supported	Once chief executives understand why the initiative is important (C) a burning platform is invoked (M) that creates a sense of urgency and commitment to change at the highest levels in local sites (O).
	In organizations with a history of short‐term projects and changing priorities (C), there is a hesitation to take a leap of faith for new initiatives (O), especially where there is perception of risk that material benefit will not be realized quickly (M).
Local adaptation supported	When an authorizing environment creates permission to make changes without worry for traditional key performance indicators (C), local site leaders have the space to reflect on discrepancies between current perceptions and desired performance (M), prompting the setting of a targets for improvement that justifies time, effort, and resource investment (O).
	Minimum standard of clinical care is clearly defined where agents in the system work out the nonnegotiables and what local improvement needs are relevant to their context (C). Balance is struck between fidelity and adaptation, allowing local sites to adjust organizational models to deliver initiatives with local skill mix, resourcing, and structures (M), allowing them to run with those that “fly” and drop those that do not (O).
Resources provided	Formal resource provision agreements (C) exert external, top‐down pressure to be accountable for the funding provided (M), leading to use of resources to drive initiative priorities rather than being absorbed into the organization budget (O).
	Mismatches between funding and need, especially uncertainty of future funding or inadequate initial funding (C), lead to difficulties in suitably staffing the initiative (issues with recruitment or local sites investing in short‐term solutions) (M) that fails to lead to sustainable change, resulting in a lack of continuity (O).
	For initiatives that are implemented without consistent enabling infrastructure across sites or that did not have a clearly defined model of care (C), variation in local hospital commitment and/or engagement with implementation support agencies (M) leads to organizations introducing unwarranted variation to match perceived local needs and local resourcing because of the emergence of many path‐dependent forms of adopting the initiatives (O).
Resources provided	
Increase ownership	Endorsement of initiative by leading clinicians (O) occurs through a process of incremental mutual adjustment in situations (M) where decision‐making authority is distributed among a mix of stakeholders (C).
Protect forward project momentum from system bottlenecks, lone saboteurs, or loss of staff	Sites that have inconsistent, absent, or unclear leadership (C) fail to transition from implementation to routine practice due to reset or lack of priorities (M), leading to a discontinuous and unstable initiative without clear and consistent direction (O).
	When the behavior of a leader clashes with other clinicians or undermines the initiative (C) and sets up roadblocks, frustration, and disillusionment toward the clinical lead develops (M), leading to lack of growth and scaling and potential for inconsistent, poor, or missed care (O).
Keep the initiative visible	Respected, credible, consistent, clear, and strong local leadership (C), which leverages preexisting personal resources, network ties, and formal and informal authority (M), leads to a more stable momentum for the initiative and trust in the leader driving it in spite of barriers such as ill‐planned or uncertain funding (O).
Coordinate processes across departments and sites and span silos	Centralized clinician roles (C) act as a conduit for standardization of care processes across organizations (M), enabling a smoother transition for patients between sites (O).
Provide relevant, authentic, timely, and meaningful data	
Mature standardized, refined data collection systems not onerous to clinicians	For data to act as a lever for clinicians to advocate for change to managers, through scaling, refining, or sustaining initiatives (O), data need to be regarded as meaningful, authentic, timely, and relevant by all stakeholders (M). Contexts that promote this are mature, standardized, refined, and piloted data collection systems; a balance of prioritized system and clinical measures triangulated with patient experience; agreement among organizations to make data visible to each other; and monitoring and evaluation frameworks accessible to clinicians (C).
	Immature data systems that lack integration with daily workflows and interoperability with legacy systems, together with a lack of staff time and skills (C), can trigger a culture of uncertainty, confusion, and risk in relation to formatively evaluating progress (M). This uncertainty results in delayed reporting and action, a disconnect from other strategic priorities, and a focus on the things that are measured rather than actual quality of care (O).
Balance system and clinical measures triangulated with patient experience data	In circumstances in which audits do not capture workflows, system barriers, and the uniqueness of local settings or there are immature communication systems between executive and frontline staff about the purpose of the audit (C), clinicians might perceive the audit as unfair or unachievable, setting them up to fail (M). This leads clinicians to focus on defending their practice rather than where things could be improved (O).
Clearly define clinical standards	When audit measures lack meaning and accuracy to local clinicians because of a lack of partnership in the audit process (C), they dismiss the audit results and rationalize the status quo (M), disengaging from the process to pursue their own priorities (O).
Trustworthy design and transparent data frameworks	Audits conducted by an external party but that is in partnership with local clinicians (C) trigger a sense of ownership and buy‐in, as clinicians recognize that the audit represents best practice (M), leading to clinicians’ trust in the process (O).
Able to demonstrate direct benefits	Early demonstration of direct benefits articulated in a way that is meaningful to clinicians (C) generates motivation that becomes reinforced through supportive feedback loops (M). The result is continued engagement and commitment to the project (O).
Support the emergence of a learning culture	
Structure opportunities for peer‐mentoring	Leaders who promote a learning culture (C) open a conduit for clinicians to engage with the auditors (M), which builds the data and external validation required to develop a convincing local case for change (O).
	Provision of infrastructure for collaboration that fosters professional networks, communication pathways, and communities of practice (C) allows clinical champions to support each other by sharing experiences, learnings, and documents that are “tried and tested” (M). This reduces duplication of effort and renewed energy to scale up solutions (O).
Activate local clinical champions	When passionate and influential clinicians (C) take responsibility for changes in response to feedback and education (M), an ongoing audit and feedback process is established (O).
Assure feasibility	Clinical champions are primed to work out how to practically apply the initiative locally (O) when they are part of a mature community of practice (C), which includes all the key players that can open doors to those with different levels of experience (M).
Develop capability and not just competency	Capability development activities and tools that address immediate clinical needs (C) open people's eyes to delivering care differently (M) and cultivating the knowledge, skill, and confidence needed to deliver the evidence‐based model of care (O).

CMO, context‐mechanism‐outcome.

### System Enabler 1: Build an Authorizing Environment

We define authorizing environment as the structures, rules, processes, and people who can grant permission or influence system‐wide change. An authorizing environment can be set by formal policies, a compelling business case for change, clearly defined priorities, and funding allocations and budgets. Less formal structures of authority include the support and interest of specific influential individuals within or external to an organization. An authorizing environment occurred in this case because the macro (NSW MoH) and mesolevels (ACI) provided legitimacy and support (e.g., resources) to the microlevel (hospitals) to make the required changes to the health care system. An authorizing environment may also be established through organizational comparison. In other words, when one hospital compares their progress to other hospitals, the comparison can legitimize the change and generate momentum. An authorizing environment facilitates change through top‐down and horizontal pressure. This authorizing environment fosters engagement and commitment to large‐scale transformation through a number of triggering mechanisms.

First, this environment gives cognitive alignment of priorities across the health system, bringing together stakeholders in a shared endeavor. A consistent and coherent message is needed across all levels—from the governing and resourcing “macrolevel” entity, the supporting “mesolevel” agencies, the executive of health districts and individual sites, through to the clinicians at the “microlevel” of the health system. In the LBVC program, “service level agreements” were negotiated with the chief executive of each health district, thus formalizing authority. The giving of permission is aimed at fostering a shared understanding of the task. This process, in turn, provides the context in which roles and responsibilities of each level can be better understood. For example, the executive group at one site kept LBVC on their agenda, obtaining regular updates and provided appropriate support and local resources. This increased clinicians’ confidence that the work was feasible. At another site, LBVC did not start on time because a key executive was leaving the position, there was skepticism over the longevity of the program based on former system changes, and the funding had been inappropriately allocated. The MoH conducted an on‐site performance evaluation and explained to the executives that the priorities were not going to change and LBVC was a permanent, long‐term change to the health care system. The new executive who took over the role, implemented the LBVC initiatives swiftly and successfully. The authorizing environment allows stakeholders to hold people accountable for carrying out their roles in the initiative, again prompting confidence and commitment. Good communication that is consistent and coherent across levels also keeps the initiative visible and on the agenda, which avoids loss of program momentum.

Second, an authorizing environment overcomes the negative results of competing priorities. When priorities are not aligned, clinicians feel they must compromise, and thus dilute their effectiveness. Change fatigue is being reported, as stakeholders struggle to meet the demands of successive or concurrent projects that pull them in different directions.^40^, ^41^ Ad hoc approaches to change set up a context of confusing priorities that can trigger disengagement, tokenistic efforts, or cynicism.

Third, an authorizing environment can include the provision of resources to enable the success of the initiative. Funding for additional or new staff, for specialist equipment or refurbishment, or for capability development activities all add to the expectation of success and feasibility of the work. This expectation, in turn, triggers buy‐in and commitment to the change. Another important resource is the active involvement of support agencies. These agencies supply expert sources of advice for the interventions (often developed by these agencies) and for practicalities of implementation.

Fourth, an authorizing environment typically permits the use of local adaptations and creativity to achieve the target of standardized clinical outcomes. This environment recognizes the unique setting of local units and understands that interventions are not one‐size‐fits‐all. Although end points of the change are well‐defined and nonnegotiable, an authorizing environment harnesses the creativity and problem‐solving abilities of clinicians to come up with adaptations to achieve the required change.

### System Enabler 2: Designate and Distribute Leadership and Decision Making

As Best and colleagues^4^ argued, large‐system transformation requires individuals at multiple levels to engage in and lead change. Effective leadership is that which is both designated and distributed such that there is shared responsibility for mobilizing efforts. Formally appointed leaders, when supported by quarantined time for change work and adequate resources, can coordinate and shape efforts. Distributed leadership shares decision making and action across implementers of change and does not rely on a single individual to lead. Distributed leadership can trigger a greater sense of ownership, giving more people a voice and an active role. Shared decision making can help build a common vision and understanding and promotes collective accountability for the project. Both designated and distributed leadership are required for large‐system transformation.

Designated leaders who hold positions that span sites or departments can provide consistency of approach and resist the introduction of unwarranted variation between sites. Such leaders also become a conduit for good ideas from one site flowing to another and span silos of experience, knowledge. and good ideas. This role can, therefore, also promote the shared vision of the initiative across levels of the system.

Distributed leadership enables the sharing of power across the group, giving responsibility and accountability to multiple individuals thus increasing ownership. For instance, a physiotherapist as clinical lead, who was widely respected within a working party group used their influence to engage other clinicians, subsequently took ownership of their assigned task and executed it successfully. Distributed leadership can mitigate the risk of having a single individual leading the initiative. It may also prevent loss of momentum if the appointed leader leaves or moves into another role. Multiple implementers who can lead concurrent activities can prevent progress from being stalled by a single leader who becomes a bottleneck. For example, a senior nurse on a ward, who was charismatic and had extensive social ties took an inappropriate shortcut in the intervention. Other staff followed suit and refused to follow best practice. Moreover, distributed leadership can mitigate the influence of an individual who may sabotage progress through lack of engagement, cynicism, or burnout.

### System Enabler 3: Provide Relevant, Authentic, Timely and Meaningful Data

Data are essential to large‐scale transformation to make the initial case for change, allow peer comparison and benchmarking, monitor progress, and evaluate outcomes. Data provided should be relevant, authentic, timely, and meaningful to the implementers. Provision of high‐quality data is key to building an authorizing environment.

Relevant data focus on indicators that the implementers can change and do not set them up to fail. For example, an indicator that measures the number of patients assessed by a podiatrist is not relevant to a site that is unable to recruit one. For data to trigger a tension for change, there needs to be a clear link between baseline data and the achievement of the best practice benchmark. Data sets developed with clinician input are more likely to be seen as authentic and relevant to care. For example, when audit measures are developed without active partnership of local clinicians, the results can lack meaning and be perceived as inaccurate by local clinicians. The presumed inaccuracy was seen to increase the likelihood of them dismissing the audit results, rationalizing the status quo, and disengaging from the process to pursue their own priorities.

Timely data recognize and factor in the impact of service constraints and pressures (e.g., bushfire emergency, COVID‐19). Meaningful data reflect processes and outcomes important to clinicians, including a balance of system and clinical measures triangulated with patient experience scores. For data to stimulate change and engagement in an initiative such as ours, there needs to be clearly defined clinical standards toward which implementers are working.

Data are best collected through mature collection systems that are tried and tested and that do not overburden clinicians. A balance is needed when designing audit measures for an initiative between local knowledge and broader‐based external knowledge. Acceptance of the data as true and authentic is heightened by internal involvement and acceptance. Data collected from across the state reinforce the alignment of priorities across the health system and build a shared vision to improve patient care. Data frameworks should be transparent and easily accessible to stakeholders from all levels of the system.

### System Enabler 4: Support the Emergence of a Learning Culture

Learning cultures are characterized by clinical teams that use data, experience, and diverse skills represented in the group to continuously improve their practice. These cultures emerge when supported by an authorizing environment and accompanying supportive resources, relevant and meaningful data, and a shared accountability that comes from a sense of ownership and agency. They can lead to the activation of clinical champions who, in a supportive environment, are encouraged to take part more actively or lead. Learning cultures can be supported by structured opportunities to collaborate with peers from other sites, so common problems can be solved together with minimal duplication of effort. For instance, we found a rural site generated solutions to overcome their barriers by listening to the problem‐solving efforts of other rural sites via online presentations. They also minimized duplication by obtaining the desired documentation from presenting clinicians. The LBVC initiative provided regular, structured peer‐mentoring workshops for leaders at the various sites, where they could learn together, drawing on the learning culture model. Interviewees that talked about this program had praise for its effectiveness. Individuals within a learning culture tend to understand and seek out opportunities to develop capability rather than just competency (i.e., the capability to respond flexibly to changing or evolving clinical situations rather than competency to manage a routine, predictable situation). Capability development activities and tools that address immediate clinical needs open people's eyes to delivering care differently: cultivating the knowledge, skill, and confidence needed to deliver the evidence‐based model of care. For instance, when junior staff were caring for patients with diabetes and complex issues, an insulin management App provided guidance. While all staff are registered and equipped to manage simple insulin/carb equations, the App supported advance skill development and increased staff confidence in managing complex interactions and to deliver evidence‐based models of care.

## Discussion

This multimethod realist study has generated a wealth of nuanced data that explain the contexts required to trigger successful large‐system transformation. It builds on the earlier work of researchers such as Best and colleagues^4^ and Levesque and Sutherland^42^ who synthesized evidence from the literature, considering the key factors that promote large‐scale change. Here, empirical data are synthesized with the literature to build a profile of four key system enablers, grounded in the experience of stakeholders from across the health system in NSW.

We have developed four system enablers: (1) build an authorizing environment; (2) provide relevant, authentic, timely, and meaningful data [equivalent to Best and colleagues^4^ simple rule 2]; (3) designate and distribute leadership and decision making [equivalent to Best and colleagues^4^ simple rule 1]; and (4) support the emergence of a learning culture.

We also found evidence of Best and colleagues’^4^ simple rule 4 to “engage physicians,” but the concept was not comprehensive, consistent, or significant across the data set. We have broadened and repositioned it under “designate and distribute leadership and decision making” (system enabler 3). Our study highlights the need to engage with all professional staff for successful large‐system transformation. Project officers and clinical leads were a mix of medical, nursing, or allied health professionals. Best and colleagues^4^ demonstrate that physicians play a significant role in health care transformation and argue for large‐scale transformation to “engage physicians.” Our research provides empirical evidence to suggest that we need to acknowledge the critical role of all health care professionals in the facilitation or hinderance of large‐scale transformation.

Similarly, we found evidence of Best and colleagues’^4^ simple rule to “attend to history,” but the concept was not consistent or significant across the data set. We have repositioned it under our novel concept the “authorizing environment” (system enabler 1).

Participants from several LHDs spoke about uncertainty—uncertainty around funding, resources, and support that would be given to the initiative. They had questions about this project's priority relative to other state and organizational initiatives. Uncertainty was reported to be a potent barrier to change, which was at odds with the known launch strategy of the LBVC initiatives. This strategy saw representatives of the MoH holding a series of meetings with LHD executives in which they delivered very clear communication around funding, priorities, and expectations, including written “service agreements.” Sites that were experiencing uncertainty turned out, relative to other sites, to have lost these transparent and clear messages. Turnover of executive staff in NSW hospitals and LHDs is known to be high,^43^ and so we surmise that, for some organizations, an adequate transfer of knowledge to incoming staff did not happen. Comprehensive handover to new staff should optimally include using clear and consistent messaging. Although the response of different people to the same messages may vary in terms of enthusiasm or ownership, consistency does reduce the possibilities of uncertainty becoming a barrier to large‐scale change.

Staff turnover has been shown to negatively affect implementation in terms of fidelity and penetration in other settings.^44^, ^45^ Turnover was explicitly acknowledged in several of our interviews as being something that could impede change and that needed to be carefully managed. Some informants discussed turnover of staff in the context of planning and conducting capacity development activities. Staff turnover, including rotation of junior medical officers and changes in leadership, resulted in a loss of knowledge and momentum to embed change. Informants spoke of the short‐term nature of junior doctor placements in each department or ward and the difficulty of progressing and then embedding improved local practices when the window for change was only a few weeks. Repeated offerings of workshops and tutorials as well as frequent updates were necessary for inpatient staff to achieve a “critical mass” that allowed new processes or enhanced understanding of disease management to become usual practice.

The four system enablers presented here should not be viewed as standalone strategies. Health system change is never linear with the many interacting and interdependent stakeholders and contextual factors that make long‐term outcomes hard to predict.^20^ Our four system enablers are designed to support one another and together build sustainable momentum for change. This reflects the understanding that when a certain context triggers a mechanism leading to an outcome, other coexisting contexts may be accelerating it or conversely working against it. Hence, positive existing contexts were seen in our data to sometimes mitigate negative ones. For example, in study sites where a learning culture was already established, even when data supplied to the team was seen as incorrect, there was still engagement and a commitment to improvement. When time is factored in, we see more complexity. For example, the outcome from one initial context may change the context in which later stakeholders operate and lead to new mechanisms being activated.^46^ Structured peer‐mentoring meetings for leaders of change from across the health districts and hospital sites resulted in an informal and self‐sustaining network of leaders that built capacity for future change.^47^

All successful large‐system changes in health care are necessarily collaborative. Stakeholders must be able to communicate with and understand each other's perspective, as their roles are inherently interdependent. A shared mental model is an organizing knowledge structure for how health care teams will interact to achieve certain tasks, which enables the coordination of actions and adaptations to match demands. This shared mental model extends to a shared understanding of how to effect change though the initiative itself—the purpose, benefits and outcomes, responsibilities and accountabilities, and a belief in its feasibility. Such a shared vision becomes a key outcome that sets up the context for progress.

Another key outcome of the synergy of the system enablers is in establishing ownership of the initiative. Successful implementation then is not a top‐down endeavor but consciously and intentionally taken up by committed individuals and teams across the levels of the system. Ownership in our study was triggered through a clear understanding of the work required, permission to make changes and adapt interventions to fit local settings, shared leadership and decision making, transparent and relevant data that highlighted areas needing improvement, and the demonstration of change and fostering a learning culture with its associated work satisfaction.

Key informants from the MoH and ACI—both agencies having a systems’ view of initiative progress and outcomes across all the NSW sites—were quick to identify sites that were “successful,” often labeling them as having a “positive learning culture.” By this they mean organizations that combine consistent engagement and action to locally generated as well as externally initiated patient improvement projects. These LHDs also developed a well‐thought–out strategy to use the allocated funding and associated resources to ensure sustainability of change. At a local level, interviewees described implementers as engaging deeply with education sessions designed to build capacity and reflecting on their practice as part of the initiative. These characteristics were fostered by the provision of resources (e.g., quarantined time, specialist educators) and the authorizing environment that accompanied the LBVC program.

Finally, we point to the reasons why the four system enablers in this study and the five simple rules in the Best and colleagues’^4^ study do not overlap more. As noted above, system enabler 2, “provide relevant, authentic, timely and meaningful data,” is equivalent to Best and colleagues’^4^ simple rule 2. System enabler 3, “designate and distribute leadership and decision making” is equivalent to Best and colleagues’^4^ simple rule 1. This study came up with two novel concepts: “build an authorizing environment” (system enabler 1) and “support the emergence of a learning culture” (system enabler 4). Best and colleagues^4^ did not identify either of these concepts, possibly because the primary studies in their realist review did not write about it.

This study did not identify Best and colleagues’^4^ simple rule 5, “include patients and families,” because patients and carers were not interview participants in this study. The interventions themselves, for each of the initiatives, had been developed with substantive input throughout from consumers and community. We relied on key informants being identified by project partners and then snowballed to other key informants from interviewees. At no point in this study were any patients or consumers identified as key informants. We did not actively seek to exclude patients and carers; rather, we judiciously followed realist sampling techniques, which led to the exclusion of patients and carers. We suggest that the participants we identified (that is, staff as the developers, supporters, and enactors of the change) are an unsurprising participant cohort given our firm focus on the implementation strategies used to try and achieve practice changes. Further research is needed to determine when and how patients and carers play a significant part in transformational change process.

### Strengths and Limitations

A strength of this study is the realist methodology. Drawing from a range of data sources, the study examined the interrelationships between contextual factors and key mechanisms driving the implementation of LBVC initiatives. Our stratification of macro, meso, and microlevels in the health system is another strength, allowing the role of these different actors to be examined. A second strength of this study is that it draws on a large and unique data set, which has enabled comparisons across multiple sites and settings over several years in various clinical conditions and services. The first limitation is the realist sampling technique, which resulted in the absence of patients and carers from this study. As mentioned above, our key informants did not identify patients and carers as key informants possibly because patients and carers were not salient change agents in their minds. Different methodologies as well as the stage of large‐system transformation under examination (e.g., design, implementation, or evaluation) impact the types of system enablers that can be generated. For example, experience‐based codesign aims to design new user‐centered services by drawing on the subjective, personal feelings and experience of users (often patients and carers) and providers (often frontline health practitioners).^48^ If the methodology is executed in line with the core principles, resulting in an accurate representation of patients and carers experiences,^49^ large‐system transformation research could foreseeably generate system enablers that speak to this cohort. The significant value of the realist approach is not under question here; rather, we wish to point to the influence of methodological choice on study outcomes.

A second limitation of this study is that it contained a relatively small number of key informants that took part in interviews. Our data collection was undertaken during the first wave of COVID‐19 cases in Australia, which limited our access to participants and necessitated video rather than face‐to‐face interviewing. These limitations influenced our participants’ capacity for engagement. We also note that data collection followed a time when NSW health services had faced catastrophic bush fires, followed by floods. Again, these external events may have affected the broader context of the LBVC initiatives.

## Conclusion

This paper identifies four system enablers that can offer implementers, whether focused on the macro, meso, or microlevel, nuanced guidance for the implementation of large‐scale health care interventions. The enablers identified—building an authorizing environment; providing relevant, authentic, timely and meaningful data; designating and distributing leadership and decision making; and supporting the emergence of a learning culture—working together to increase ownership and feasibility of change. Although the current study is based in Australia where the focal sites were public hospitals, results are based on program theories and, therefore, provide a sound model for change. With concerted application, these findings can pave the way not just for a better understanding of greater or lesser success in intervening in health care settings but ultimately to contribute higher quality, higher value, safer care.

## Funding/Support

This work was supported by the Medical Research Future Fund (APP1178554, CI Braithwaite). The funding arrangement ensured the funder has not and will not have any role in study design, collection, management, analysis, and interpretation of data, drafting of manuscripts and decision to submit for publication.

## Conflict of Interest Disclosures

The authors have no conflicts of interest to declare.

## Author Contributions

Emilie Francis‐Auton: . Mitchell N Sarkies: . Natalie Roberts: . Johanna Westbrook: . Jean‐Frederic Levesque: . Rebecca Hardwick: . Peter Hibbert: . Chiara Pomare: . Jeffrey Braithwaite:

## Supporting information

Supporting Material

Supporting Material
