# Supplementary material for: Four System Enablers of Large‐System Transformation in Health Care: A Mixed Methods Realist Evaluation
Source: Milbank Q. 2023 Dec 25;102(1):183–211. doi: 10.1111/1468-0009.12684 (PMC10938932; doi:10.1111/1468-0009.12684)
Supplement: Supplementary file 1 — Supporting Material [file MILQ-102-183-s001.pdf]

Interview schedules

Semi-structured interview schedules are specific to each initial program area, matched to key informants of each area. Questions drilled down into mechanisms (M), specific contexts (C), and outcomes (O). Questions were directly linked to proposed initial program theories expressed as CMO statements.

Not all questions were asked as we were trying to elicit rich and highly nuanced data about CMOs. Interviewers were responsive to the information interviewees provided, drilling down on contexts, mechanisms and outcomes as they were mentioned.

All participants

Thank you for agreeing to participate today.

I've started recording, I just wanted to check you have read and understood the information form and are happy to participate today?

Purpose of this interview is to understand more about the implementation of the LBVC program, so this is not an evaluation and we're not evaluating you personally. We're trying to understand how the various implementation strategies led to either planned or unplanned changes throughout the system.

Have you participated in a research interview before? The interview style we are using is a bit different to what people might be used to, I'll be asking a lot of probing questions, such as 'which specific LHDs? How do you know x or y" – but just to reassure you that when you name specific people or sites that it is all confidential.

Can you briefly describe your role in relation to the LBVC program?

Specific questions for each initial program area:

Business case for change

I'd first like to talk about the business case for change and the role that played in the implementation of LBVC. We've got a bunch of ideas about that, some of which I'll introduce a bit later, but can you tell me initially what's involved in a successful business case for change?

How was the business case for change received here? (M)

Was there a difference between how staff felt the hospital was performing and what the data said? (M)

Did that lead to a bit of myth busting at your hospital? (M) *Prompt: Did that change mindsets? (M)*

Did that lead to challenging the status quo? (M)

What did that lead to? (O) *Prompt: was a benchmark set to keep this site accountable (O); Did that benchmark help to justify time, effort and resource investment? (O)*

What was it about [insert LHD/initiative] that allowed that to occur? (C) *Prompt: did the program designers create an environment where you had the space, tools, information and permission to make changes without concern about being evaluated? (C) Prompt: can you give me an example? (C)*

Now talking about resistance:

Was there any initial resistance to the LBVC program and the change it would bring to this hospital? (O)

Could you give me an example? (O)

Was resistance to change obvious or under the surface? (M)

Why do you think there was that resistance? (M)

Was the resistance to change overcome here? How? (M)

Do you have any other examples? (M)

What influenced this resistance to change? *(C) Prompt: was there leadership turnover that changed the organisations vision/priorities? (C) Prompt: were there any doubts about there being sufficient evidence for the initiatives? Prompt: was there disagreement about the financial sustainability of the program? (C)*

Did you have any involvement in the setting up or maintenance of local data reporting for the tranche one LBVC initiatives? (O)

Are the models of care generally endorsed? (O)

Who by? (O)

What about the measures? (O)

Who by? (O)

Who else is really aware of the measures getting reported? (O)

Did this help set up a roadmap for change? (O)

How was this endorsement/consensus achieved? (M)

Did people have to adjust to the needs and requirements of other stakeholders? (M)

Can you please give an example? (M)Did the mix of decision makers influence the endorsement of measures? (C)

Was there a different mix of decision makers for certain initiatives? (C) Do you have any examples? (C)

Why did this mix of decision makers' work for each initiative? (M) *Prompt – Did certain groups have more say than others? (C) Prompt – Was there a mix of generalists and specialists? (C)*

Is the current data reporting adequately capturing local successes for each initiative? (O)

What did this lead to? [e.g. disengagement] (O)

Why/why not? (M)

Do you think a change in measures or a change in approach is needed for any initiatives? (O)

Can you please give an example? (O)

Why didn't the approach change? (O)

Data monitoring and evaluation

We've heard that there might not really be a true consensus around the initiatives, patient cohorts and measures collected, what are your thoughts on this? (M) *Prompt: did the rush to get these initiatives up and running impact this consensus building? (M)*

What is it about these initiatives that makes it difficult to measure their success? (C) *Prompt: we've heard them described as "wicked problems" without really well-articulated solutions. (C) Prompt: have there been disagreements between clinicians about the specifics of care (e.g. generalists vs. specialists)? (C)*

How is the monitoring and reporting of LBVC generally perceived locally? (M)

Are the measures considered authentic? (M)

Are the measures considered relevant? (M)

We've heard that when measures are regarded as authentic and relevant, they can act as a lever for change when clinicians are advocating for funding or other support from middle managers. Is that reflected in what you've seen? (M)

Why [or not] is this the case? (M)

Can you give an example? (M)

How are the reported measures acted upon? (O) *Prompt – Establishment of governance and implementation processes? (O) Prompt – Realignment of initiatives? (O)*

Are there examples of this we can draw from? (O)

Bringing this back to what we were talking about earlier, what makes measures authentic and relevant to clinicians or others at your LHD? (C) *Prompt: mature monitoring and evaluation systems with a balance of system, clinical and patient measures? (C) Prompt: simple, actionable documents (e.g. ACI two-pagers)? (C) Prompt: data visable between hospitals and LHDs? (C)*

Data collection systems built into day to day workflows rather than just another task? (C)

Were there any perceptions of risk, uncertainty or confusion related to the data monitoring and evaluation? (M)

Why was that the case here? (M)

What was the consequence of this uncertainty and risk? (O) *Prompt – Delayed reporting and acting on data? (O) Prompt – Was there any scope creep and escalating costs here? (O)*

What influenced this? (C) Prompt – Systems still under development (e.g. HOPE)? (C) *Prompt – Integration with daily operations and interoperability with existing systems? (C) Prompt – Limited staff time and skills? (C)*

Are any of the tranche one initiatives considered a success locally? (O) *Prompt: if so, then how did you establish a track record of success? (M)*

Was an early demonstration of benefit used to motivate clinicians/administrators? (M)

How do you know? Can you provide an example?

What influenced this? (C) *Prompt: if not, then how was it perceived locally? (O)*

Is it seen as performance management or as an improvement process? (O)

How do you know? Can you provide an example?

What influenced this? (M/C)

Were there issues with fidelity to the models of care here? (O)

Why do you think that was? (M)

Did you have access to enough formative feedback to respond or adjust? (M)

What else influenced this? (C)

Were there any issues with communication of how the initiatives were going here? (C)

Resource Provision

Can you please describe the resources provided to your LHD for LBVC? *Prompt: Financial? Headcount? Infrastructure? Equipment?*

How were those resources used to implement the different initiatives? (O)

Did this change over time? (O)

How did this change over time? (O)

Were certain initiatives funded better than others? (O)

What were the main things that influenced how resources were used? (M)

Was the level of accountability shared between your LHD and the Ministry? (M)

How did this impact funding locally? (M)

Can you please give an example?

How did the broader environment surrounding LBVC impact the use of resources? (C) *Prompt: Endorsement and priority given by the Ministry? (C) Formal agreements? (C)*

Were any resources absorbed into the organisation bottom line? (O)

Was the funding for the program sustainable? (O) *Prompt: stopping and starting? (O)*

What influenced this to happen here? (M) *Prompt: was the sustainability of the program ever questioned? (M) Prompt: how was the upfront funding perceived? Was it seen as a "gift" from the Ministry? (M) Prompt: was the funding considered as a large, one-off payment? (C) Prompt: did this effect resource allocation at your LHD/initiative? (M) Prompt: did inconsistencies in funding between initiatives impact the implementation? (C)*

Tight-tailor("loose")-tight approach

Do you think a balance was struck between fidelity to the models and local adaptation? (O)

How was this balance achieved? (M)

How do people work out the non-negotiables from the parts that can be changed? (M)

Did your LHD prefer more discretion or more structure? (C)

Can you give me an example? Why was that? (M)

Were there certain initiatives where greater discretion or structure worked better? (C)

Why was that so? (M)

Can you give me an example? (M)

Did that discretion or structure help when there were fires/floods/COVID? (C)

Is there a risk that the lack of direction for implementation created inefficiencies (e.g. having to reinvent the wheel – job description for podiatrist)? (O)

What was the outcome of this? (O)

Did the process of localising initiatives ever create unwarranted variation? (O)

How much did you tailor the initiatives? Can you give me an example?

Why did this happen? (M)

What did that lead to? (M)

What was it about xy? (C) Was there existing infrastructure for those initiatives? (C)

How were successful processes and structures for implementing LBVC shared with your LHD?(O)

What was shared? (O)

Why did that help? (M)

Did your LHD gain access to additional resources through engagement with ACI? (M)

Did ACI have a role in legitimising approaches locally? (M)

Can you give me an example? (M)

Was the model of care appropriate to the context and applicable? (C)

Was ACIs support tailored to your site? (C)

Did you require more or less support than was provided from ACI? Why was that? (C)

Did your LHD require additional guidance because of the size of this program? As in, was it considered too large to control? (O)

What was the consequence of this large scale? (O)

Were local opportunities for innovation missed? (O)

What was it about the size and scale that lead to these outcomes? (M)

Can you give me an example? (M) Did the project lead or clinical lead have knowledge or relationships to make things happen?

What was the real impact of the lack of clarity around how to implement the initiatives? (C) Was that lack of clarity around the roles of ACI and the ministry OR about the core models of care?

Capability development

I'd first like to talk about the capability development components for LBVC and the role that played in implementation of the program. We've got a few ideas about that, some of which I'll introduce later, but can you tell me initially what made up the capability development activities locally? Prompt: How did that help to drive change? (M) What did that lead to? (O) How did you find out about that? Was that the case for everyone? (C)

Do you think that clinician's knowledge and skills have improved as a result of these activities? (O)

Did that contribute to the success of the initiative/s? (O)

How do you know?

Do you think it was effective in driving change because the staff were all on the same page? (M) *Prompt: Did it build confidence?(M) Did it build self-esteem? (M) Did it focus clinician's attention? (M)*

How was the training made relevant and accessible to clinicians at your site? (C) Prompt: Was it because the training addressed immediate need? (C)

Was there enough knowledge and skill developed? (O)

Why was this? (C)

What did that lead to? (M)Could you give me an example?

Do you have any other examples?

Were there any examples where high staff turnover led to repeated mistakes? Prompt: Reinventing the wheel? (M)

How was it overcome, if it was?

Do you think your site adopted a quality improvement culture in relation to the LBVC initiatives? (O)

What does that look like? (O)

How did that happen there? What came before? (M)

Were staff given rostered time for the initiatives? (C)

Did staff have enough tools or activities? (C)

How did that help? (M)

Did the knowledge and skills development ever not translate into practice change? (O)

Why was that? (M)

Which initiatives? (C)

Were the activities ever considered an impost on clinician's time? (M)

Which activities? (C)Who was involved in developing the training? (C)

Did it match the clinician's needs? (C)

Were clinicians involved in the development? (C)

Were the LBVC initiatives a community-wide priority in your LHD? (M)How did they do that? (M)

What did that lead to? (O)Did it enhance the reach and scale of the program? (O)

Why did people outside the hospital get on board? (C)

How did [answer] lead to a community wide priority?

Were there peer interaction activities? (C)

Did you share or receive information about what worked or didn't work from other sites? (M)

What did this lead to? (O)

Did the lack of information flow prevent success at your site? (O)

What did you miss out on? (what information?) (C)

Was there peer learning, mentoring, ongoing communities of practice? (C)

Collaboration

I'd first like to talk about the role of collaboration in the implementation of LBVC. We've got a bunch of ideas about that, some of which I'll introduce a bit later, but can you tell me initially what's involved in successful collaboration between not only organisations but also individuals?

Were you involved in any of the communities of practice developed for LBVC? *Prompt: peer mentoring workshops, clinical networks, monthly project lead meeting.*

What does that lead to? (O) *Prompt: key stakeholders linking up independently to support implementation? (O) Prompt: new professional networks? (O)*

Do you have any examples? (O)

Why does it work like that? (M) Prompt: informal, face to face networking? (M) Prompt: clinical champions ready to implement initiative? (M) Prompt: equal access to support network? (M)

Do you know whether it worked differently at other hospitals? (M)

How did ACI engage with you prior to implementation? (C) Prompt: how did you (why didn't you) get that informal, face to face networking? (C) Prompt: what are the ingredients for [insert mechanism] getting clinical champions ready for implementation? (C)

Now onto some of the other collaboration and implementation support (both LHD and ACI).

Within your LHD and between ACI, how did you know who was responsible/accountable for what? (O) Prompt: was there an awareness of the respective roles of other teams and team members? (O) Prompt: how was an environment created around this initiative so that is had some formal legitimacy? (O)

What was needed before to ensure that [insert outcome] occurred? (M) *Prompt: what was ACI's role here? (M) Prompt: did the support from ACI match the needs of you LHD? (M)*

Can you give me an example of ACIs support matching (or not) your needs? (M)

What were people's previous experience running these types of initiatives? (C) *Prompt: was there uncertainty around who was responsible for what? (C)*

Audit and feedback

Do you believe that clinicians trust the audit process? (O) Could you give me an example?

Do you think the measures contributed to capability development? (O) Could you give me an example?

Has that always been the case? (C)

Does trust come from staff having been involved in the audit measures? (C)

Why is that the case? (M)

We've heard that when people feel they have a say/buy in in developing audit measures, they have more trust [or O2] in those measures. Has that been the case? (M) Why was that? (M)

Were any staff disengaged from the process? (i.e., did they do their own thing/use their own measure) (O) Why was that? (M) *Prompt: Were the measures not useful? Did they lack meaning and accuracy? Were they considered an impost?*

Was it because the tools were not created in partnership with clinicians? (C) Prompt: Were there too many variables? (C) Unclear/conflict/absent evidence? (C) System barriers to delivery? (C)

Do you think there was a learning culture around the measures? (C) What does that mean/look like here?

Was there someone at your site who flagged that the audit was going to happen?/Organised ACI's engagement?

If yes – And do you think that the audits provided clinicians with support for their case for change? (O)

Did it provide a sense of external validation? Can you give an example? (M)

We've heard it said that when people are able to integrate audit and feedback with their local experiential knowledge it really helps – was that possible anywhere? (M)

What's your experience been of that? (M)

In terms of how it helps, what would you call that? (M)

We've called it sensemaking (then share mechanism). Does that fit with your understanding?

Do you think the audit measures adequately captured local workflows? (C) *Prompts: Immature systems? (C) Lack of understanding? (C)*

If no – why not?

Were the audit measures perceived as unfair? (M) *Prompt: Unachievable (set up to fail)? (M)*

What did that lead to? (O) Prompt: Did they take it as an attack on their professional integrity? (O) So was there more of a focus on integrity rather than patient care experience/

If yes: How did this impact clinicians' perceptions of the audit and feedback process? (O)

What motivates clinicians to change or improve practice? (O) What else? (O) Can you give an example?

Did this ever lead to considering other ways of working? (M)

Any competition with other sites or people locally? (M)

What influenced that to happen? (C) *Prompt: Did you hear about the results of other sites?*

Was there agreeance among the clinicians here regarding best practice principles of care?

Was there any resistance to the feedback? (O) *Prompt: Was it perceived as a threat to clinical autonomy? (M)*

Could you give an example?

How did the clinical leaders respond to the audit and feedback? (O)

Did you develop a local improvement plan? (O) Can you tell me about that plan…

Would you say that was in response to the audit and feedback? (O)

What prompted the plan being developed?

Was it because leaders took accountability/responsibility for the audit? (M)

Did you get all the right people in the room when ACI presented the results? (C)

We've heard that it's important that feedback goes to the people with influence to make changes, as it can lead to improvements. Was that the case here? (C) Could you give me an example? Who was the person of influence?

Did you think this was going to be replaced with a new initiative, or here to stay? (O) *Prompt: history of churn; initiatives seen at temporary*

If you made a plan, was it followed through or was it a bit tokenistic? (O) *Prompt: gaming the measurement process*

Why was that? *Prompt: Was it because the feedback wasn't appropriately contextualised for your site? (M) Prioritised?*

Why was the feedback not contextualised? *Prompt: Was it because the person who delivered the feedback did not have an understanding of the local setting? (C)*

Was there enough time to digest the information before making improvement decisions? (C)

Leadership

I'd first like to talk about leadership and the role that played in the implementation of LBVC. We've got a bunch of ideas about that, some of which I'll introduce a bit later, but can you tell me initially what's involved in a successful leadership for a program like this?

Do you think the initiatives had a high profile and visibility locally? (M)

Why do you think that was? (M)

What did this lead to? (O)

Did it lead to senior executives getting more involved?

Did your LHD work closely with ACI or MoH? What was their role in promoting the visibility of project locally? (C)How was this achieved? (C)

What did this lead to? (M)

Do you have any examples?

Were there any examples where the initiatives were siloed between departments? ? (O)

Why was that? (M)

What did that lead to? (O) *Prompt: burnout?*

Why else were they siloed?

What was the precursor to this [outcome- siloing or burnout]? (M)

Was that because there was a lack of local authority/executive sponsor [no one] to join the dots? (M)

How did that lead to siloing/burnout? (M)

Can you give any examples?

How were the initiatives viewed there? (C)

Was there any other reason why the executive sponsor or clinical leads weren't so actively involved there? (C)

Do you feel there was momentum for the initiatives here? (O)

Why do you think that happened here? (O)

Is there trust in the clinical leader here? (O)

How did X [the Clinical leader] make things happen? (M) *Prompt: Formal and informal authority? Personal networks? (M)*

Can you give any examples?

What was it about this site which facilitated the leader to make things happen? (C)

Were there generally the same people involved in LBVC or was there high staff turnover? (C)
What did that consistency (or inconsistency) in leadership lead to?

Did the initiatives become "routine practice" here? (M)

If not, why didn't that happen? (M) (e.g., change in leadership? And following that, a reset of priorities?)

What did that lead to? (O)

Halts in the initiative? (O)

Lack of clear direction? (O)

What was it about [example] that prevented the initiative translating to routine practice? (C)

Prompt: Unclear leadership? or absent leadership? Inconsistent leadership? (C)

Did clinicians take a lead on the initiatives even when it wasn't formally part of their role? (O)

Can you give any examples of this?

How did this happen? (M) *Prompt: Collective motivation, inspiration, high levels of morale, trust, respect, admiration? (M)*

What was it about those leaders that fostered [mentioned above] (C)

Do you have any examples?

Do you have any examples where this clinical leadership didn't work? (O)

What was the result? (O)

Why there? (M)

What was it about that clinical or nonclinical leader? (C) *Prompt: characteristics – authoritarian, lazy, etc.*
