# Supplementary material for: Four System Enablers of Large‐System Transformation in Health Care: A Mixed Methods Realist Evaluation
Source: Milbank Q. 2023 Dec 25;102(1):183–211. doi: 10.1111/1468-0009.12684 (PMC10938932; doi:10.1111/1468-0009.12684)
Supplement: Supplementary file 2 — Supporting Material [file MILQ-102-183-s002.pdf]

MRFF FINAL CMOs

AUDIT AND FEEDBACK

Audit and feedback involves providing healthcare professionals with performance feedback on specific target indicators in order to prompt changes to improve their practice (1).

IPT 1 – Ownership and Buy In

C: Audits conducted by an external party but in partnership with local clinicians, to ensure staff have input into the process.

M: Triggers a sense of ownership and buy in, as clinicians recognise that the audit represents best practice.

O: Trust in the process and capability developed for future audits conducted locally.

CMO: Audits conducted by an external party but in partnership with local clinicians, triggers a sense of ownership and buy in, as clinicians recognise that the audit represents best practice. Leading to trust in the process and capability developed for future audits conducted locally.

IPT 2 – Rationalising

C: Lack of partnership in audit process: Large number of audit variables used, wrong cohorts audited, unclear, conflicting or absent evidence for audit measures, system barriers to care delivery outside of clinicians control. Measures lack meaning and accuracy are considered an impost.

M: Staff dismiss the audit results and rationalise the status quo.

O: Disengage from the process and pursue their own priorities from other means of performance measurement and existing practices.

CMO: When audit measures lack meaning and accuracy to local clinicians due to a lack of partnership in the audit process, they dismiss the audit results and rationalise the status quo, disengaging from the process to pursue their own priorities.

IPT 3 – Sensemaking

C: Local sites with leaders who promote a learning culture, open a conduit for clinicians to engage with the auditors and lead the development of improvement plans.

M: Sensemaking: local clinicians are open to hearing about their performance against measures, and on reflection are able to integrate this information with local, codified knowledge and evidence by proxy, to make sense of the implications for those receiving care.

O: External validation of case for change: evidence for implementing changes is provided to clinicians to support their case for local site improvements and educational requirements.

CMO: Leaders who promote a learning culture open a conduit for clinicians to engage with the auditors, which builds the data and external validation required to develop a convincing local case for change.

IPT 4 – Unfairness and Integrity

C1: Audits do not capture local workflows and/or system barriers and/or the uniqueness of local settings

OR

C2: Immature communication systems between executive and frontline staff for managing expectations and understanding of the implementation support agencies role (clinicians can misinterpret the audit as a performance management process rather than a learning opportunity).

M: Clinicians perceive the audit as an unfair and unachievable process that sets them up to fail.

O: Focus on defending current practice rather than where things could be improved.

CMO: In circumstances where audits do not capture workflows, system barriers and the uniqueness of local settings or there are immature communication systems between executive and frontline staff about the purpose of the audit, clinicians might perceive the audit as unfair or unachievable, setting them up to fail. And this leads them to focus on defending their practice rather than where things could be improved.

IPT 5 – Competition

C: Informing clinicians of best practice care from an external source.

M: Facilitation from an external incentive, peer competition or credible source.

O: Can overcome external locus of control and trigger motivation to improve or maintain performance.

CMO: Informing clinicians of best practice care from an external source does not trigger an intrinsic motivation to change or maintain performance, unless facilitated by an external incentive, peer competition or credible source.

IPT 6 – Responsibility and Accountability

C: Feedback and education reinforced at the point of care to passionate people who can influence practice change.

M: Responsibility and accountability: clinicians assume responsibility for audited components of care.

O: Audit and feedback becomes an ongoing process and is leveraged to gain managerial support for improvement activities.

CMO: When passionate and influential clinicians take responsibility for changes in response to feedback/education, an ongoing audit and feedback process is established and can be used to gain managerial support for improvements.

IPT 7 – Tokenistic Plan

C: Feedback delivered by an outsider without sufficient time provided to digest information before making improvement decisions or specific outline of support that could be provided.

M: Feedback does not provide a meaningful foundation for quality improvement.

O: Local hospitals continue working on their own improvement priorities.

CMO: Feedback does not form a meaningful foundation for quality improvement when it is delivered by an outsider without sufficient time for interpretation or clear outline of support being offered. In response, local hospitals continue working on their own priorities.

IPT 8 – Threat to Autonomy

C1: Rigid criteria used for audit rather than broad principles of care and not localised to audience
C2: Audit and feedback delivered to medics by non-medical professional (e.g. community nurse, project officer, ect.)
M: Threat to autonomy: Clinical leaders perceive feedback as a directive and are frustrated that their expertise is not respected.
O: Resistance: Feedback and proposed changes are resisted or not engaged with because clinicians feel like measures do not adequately capture their work.

CMO: Rigid criteria for audit rather than broad principles of care and feedback is delivered to medics by non-medical professionals leads to clinical leaders perceiving feedback as a directive and are frustrated that their expertise is not respected. This leads to feedback and proposed changes being resisted or not engaged with because clinicians feel that measures do not adequately capture their work.

BUSINESS CASE FOR CHANGE

A business for case for change clearly sets out evidence and information for those overseeing the initiative to allow them to decide whether to support a proposed project before significant resources, including time, are committed to its development (2).

IPT 1 – Burning Platform

C1: Program designers create an "authorising environment" which carves out space and provides the tools, information and permission to make the change without worry for traditional key performance indicators.
M1: Myth busting, re-framing and unfreezing current mindsets: demonstrating through data a discrepancy between current perceptions and desired performance creates dissatisfaction with the status quo.
O1: A target or benchmark is set to keep local site accountable, prioritising the justification time, effort and resource investment.

CMO1: When an authorising environment creates permission to make changes without worry for traditional key performance indicators local site leaders have the space to reflect on discrepancies between current perceptions and desired performance, prompting the setting of a targets for improvement that justifies time, effort and resource investment.

C2: Chief executive officer understands the "why" and expresses support locally: credible evidence-base used, professionally packaged with problem contextualised, and magnitude of cost implications presented.
M2: Case for change, through demonstration of better clinical and financial outcomes, is invoked, creating a sense of necessity.
O2: Creating commitment to change from the highest levels: necessity for change is accepted as urgent and mandatory.

CMO2: Once chief executives understand 'why' the initiative is important a 'burning platform' is invoked that creates a sense of urgency and commitment to change at the highest levels in local sites.

M3: Social influence and peer pressure to get on board with the secretary's priority.

IPT 2 – Overt or Covert Resistance

C1: Executive-level - Leadership changeover and competing priorities/relative importance of the initiative.

OR

C2: Executive-level - Misunderstanding or disagreement about the financial sustainability and implementation of the program

OR

C3: Clinician-level - Lack of transparency for decisions made at the executive-level that affect clinicians.

M: Overt or covert resistance: Counterargument against case, confusion about priorities, superficial agreement or indifference, open debate amongst stakeholders followed by covert resistance.

O: While the initiative can remain valued within different parts of the organisation, operationalisation becomes problematic under these circumstances.

CMO: Overt or covert resistance to the initiative is cultivated at the clinician-level when there is a lack of transparency regarding decisions that affect them and at the executive-level in situations with high leadership turnover, changing priorities, and uncertainty around the financial sustainability. While the initiative can remain valued within different parts of the organisation, operationalisation becomes problematic under these circumstances.

IPT 3 – Consensus Building

C: Case for change tailored to different audience and existing organisational priorities.

M: Consensus building: Genuine consensus between those advocating for change and those responsible for change is developed through an understanding of the problem before diving in with solutions.

O: Shared vision and resolve: A shared vision and resolve is arrived at allowing the seamless integration of initiative into existing organisational structures, performance indicators, and activities.

CMO: A case for change that is tailored to different audiences/priorities enables genuine consensus building to take place between those advocating for the initiative and those responsible for delivering care, which builds a shared vision for the seamless integration into existing organisational structures, performance indicators, and activities.

IPT 4 – Risk Aversion

C: Previous negative experiences with change efforts.

M: Believe in future-orientated direction of program but there's a perception of risk because material impacts are not seen quickly.

O: Efforts and investments made, but hesitant to take leap of faith because benefits might be more conceptual and not realised in organisation bottom line.

CMO: In organisations with a history of short-term projects and changing priorities, there is a hesitation to take a leap of faith for new initiatives, especially where there is a risk that material benefit will not be realised quickly**.**

IPT 5 – Self Organisation

C: Senior leadership signalling and strategic communications to ensure all actors in the system understand that the program is to become business as usual.

M: Self-organisation: local sites and individuals reprioritise depending on local needs and personal priorities, and responding to feedback from other sites, creating order at the system-level from initially disorganised efforts.

O: Change valence at the Health service- or personal-level leads to the establishment of sponsorship, governance, leadership structure, and multidisciplinary workforce to support improvements.

CMO: Local sites make changes to their sponsorship, governance, leadership structures which then leads to self-organisation at the system level when it's understood that the initiatives are to become business as usual.

CAPABILITY DEVELOPMENT

Developing capability involves moving beyond clinical competency in dealing with routine clinical situations, to the ability to respond flexibly, navigate change, and adapt to unpredictable or rapidly changing circumstances (3-5).

IPT 1 – Making it Relevant

C: Easy access to capability development activities and tools that are pragmatic and relevant to immediate clinical needs.

M: Creates a shared mental model for what is important/what to focus on, by opening people's eyes to seeing things differently.

O: Knowledge, skill and confidence to deliver evidence-based model of care is cultivated throughout the organisation over time, which supports the replication of successes across sites.

CMO: Capability development activities and tools that address immediate clinical needs open people's eyes to delivering care differently, cultivating the knowledge, skill and confidence needed to deliver the evidence-based model of care.

IPT 2 – Investment in Quality Improvement

C: Investment in quality improvement and safety: In situations where staff value the need for the training and managers actively facilitate participation.

M: Accountability: Executive team and clinicians each understand each other's responsibilities and accountability is established between staff.

O: Adoption of QI culture: Engagement with capability development resources increases.

CMO: Executive team and clinicians understand each other's responsibilities and engagement with capability development resources increases, in situations where the need for training is valued and participation is actively facilitated.

IPT 3 – Turnover and Capability Loss

C: High staff turnover: Organisations with high staff turnover, particularly of key stakeholders for the initiative.

M: Knowledge loss: Loss of knowledge prevents workforce from learning from experience, repeating mistakes, reinventing the wheel, and inhibiting continuous improvement.

O: Incoherence: Failure to produce critical mass of knowledge and skills within organisations workforce to consistently deliver evidence-based model of care.

CMO: Organisations with high staff turnover, particularly of key stakeholders for the initiative, leads to a loss of knowledge that prevents the workforce from learning from experience, repeating mistakes, reinventing the wheel, and inhibiting continuous improvement. This results in a failure to produce critical mass of knowledge and skills within organisations workforce to consistently deliver evidence-based model of care.

IPT 4 – Community-Wide Priority

C: Centralised clinical lead role across organisation.

M: Clinical leads acts as a conduit for standardisation of care across sites.

O: Reach and scale: The success of initiatives can be replicated across multiple sites at scale allowing a smooth transition for the patient across sites.

CMO: Centralised clinician roles act as a conduit for standardisation of care processes across organisations enabling a smoother transition for patients between sites.

COLLABORATION

Collaboration enables social processes within and across local implementers such as shared decision-making, learning together, supervisory and peer mentoring activities and interdisciplinary working. This helps to develop a shared understanding of what the new initiative means, defines how tailoring and adaptation to local conditions is to be done, and builds local capacity to achieve the change.

IPT 1 – Structuring peer-mentoring opportunities

C: Provision of infrastructure for collaboration that fosters professional networks, communication pathways, and communities of practice.

M: Clinical champions support each other by sharing experiences, learnings and documents that are "tried and tested".

O: Reduced duplication of effort and renewed energy to scale-up solutions.

CMO: Provision of infrastructure for collaboration that fosters professional networks, communication pathways, and communities of practice allows clinical champions to support each other by sharing experiences, learnings and documents that are "tried and tested". This reduced duplication of effort and renewed energy to scale-up solutions.

IPT 2 – Skilful external facilitation and support

C: Uncertainty surrounding lines of accountability.

M: Tailored support is needed to manage relationships and align conflicting information and priorities from difference sources.

O: legitimises and authorises initiative activities, improving situational awareness of who is responsible for what.

CMO: In situations where there is uncertainty re. lines of accountability, tailored support is needed to manage relationships and align conflicting information and priorities from difference sources. This external support helps to legitimise and authorise initiative activities, improving situational awareness of who is responsible for what.

IPT 3 – Activating clinical champions

C: Community of practice matured and includes key players (Gurus) who can open doors to those with different experiences.

M: Priming of clinical champions and other key influencers with strong local voice to have confidence through both formal and informal networking.

O: Clinical champions work out how to practically apply the model locally through a process of self-organisation.

CMO: Clinical champions are primed to work out how to practically apply the initiative locally when they are part of a mature community of practice, which includes all the key players that can open doors to those with different levels of experience.

IPT 4 – Shared decision making

C: Conscious efforts made to remove hierarchy between professions and clearly define the purpose of peer mentoring networks as a solutions oriented, supportive and inclusive culture.

M: Common understanding of everyone's contributing roles to delivering the initiative, solve problems and do things better.

O: Person-centred, collective action is taken to deliver and refine the initiative (less focus on role delineation).

CMO: A common understanding of what is needed to deliver the initiative prompts collective action when conscious efforts are made to remain solutions oriented and remove hierarchies from peer mentoring activities.

DATA MONITORING AND EVALUATION

Data monitoring and evaluation provide implementers across all levels of the health system (government, support agencies and hospital-based clinicians) with objective, benchmarked evidence of the effectiveness of their activities or the need to address particular areas, sustaining focus and momentum on the program.

IPT 1 – Incremental Mutual Adjustment

C: Decision making power distributed between a mix of stakeholders (e.g. specialists, generalists, different professions) who already have some experience in similar pilot projects.

M: Prioritisation of initiative selection through a process of incremental mutual adjustment.

O: Endorsement of guideline or model of care, roadmap for change, and measurement of progress by leading clinicians.

CMO: Endorsement of initiative by leading clinicians occurs through a process of incremental mutual adjustment in situations where decision making authority is distributed between a mix of stakeholders.

IPT 2 – Dig Their Heels In (Reluctance to Change Midstream)

C1: A "wicked problem" without a well-articulated solution, creating competing agendas for data collection and how to reach evidence thresholds.

OR

C2: LHDs consider models too prescriptive and unable to implement at their site (e.g. mandatory staff not available in rural area)

M: A false consensus is created through a peremptory approach to the selection of initiatives, definition of cohorts and selection of measures.

O: MoH and ACI were reluctant to change cohort or measurement approach in spite of clinicans' concerns, leading to some LHDs disengaging and others creating workarounds to show success without strictly adhering to the model.

CMO: A false consensus or peremptory approach arises when a wicked problem without a well-articulated solution or a model that is too prescriptive / not feasible. This results in MoH and ACI being reluctant to change the cohort or measurement approach in spite of clinicans' concerns, leading to some LHDs disengaging and others creating workarounds to show success without strictly adhering to the model.

IPT 3 – Authenticity and Relevance

C1: Mature, standardised, refined and piloted data collection systems.

OR

C2: A balance of prioritised system and clinical measures triangulated with patient experience.

OR

C3: Systems treated as sociotechnical change rather than delivering a product.

OR

C4: Organisations agree to make data visible to each other.

OR

C5: Monitoring and evaluation frameworks accessible to clinicians.

OR

C6: Simple 2-pager documents describing different ways the initiatives can be implemented, rather than statements of principles.

M: Data regarded as meaningful, authentic, timely and relevant by all stakeholders, and used to demonstrate benefits or highlight where improvements could be made.

O: Data acts as a lever for clinicians to advocate for change to managers, through scaling, refining or sustaining initiatives.

CMO: For data to act as a lever for clinicians to advocate for change to managers, through scaling, refining, or sustaining initiatives, data needs to be regarded as meaningful, authentic, timely and relevant by all stakeholders. Contexts which promote this are: Mature, standardised, refined and piloted data collection systems; a balance of prioritised system and clinical measures triangulated with patient experience; organisations agree to make data visible to each other; monitoring and evaluation frameworks accessible to clinicians.

IPT 4 – Uncertainty

C1: Complex, immature and untested centralised data systems that do not capture local nuances in data.

OR

C2: Lack of integration with daily operations and interoperability with legacy systems.

OR

C3: Limited staff time and skills.

OR

C4: Technology push rather than sociotechnical change.

M: Culture of uncertainty, confusion, and risk in relation to formatively evaluating progress.

O1: Delayed reporting, interpretation and action based on data, as well as scope creep.

OR

O2: Surveys cumbersome to administer and findings difficult to interpret.

OR

O3: Disconnect from other strategic priorities.

OR

O4: Focus is placed on the things that are measured rather than actual quality of care.

CMO: Immature data systems lacking integration with daily workflows and interoperability with legacy systems, together with a lack of staff time and skills can trigger a culture of uncertainty, confusion, and risk in relation to formatively evaluating progress. This uncertainty results in delayed reporting, and action, a disconnect from other strategic priorities and a focus on the things that are measured rather than actual quality of care.

IPT 5 – Motivation

C1: Well-articulated and defined initiative and early demonstration of direct benefit

OR

C2: Ongoing engaging and supporting feedback loop established that facilitates conversations between administrators and clinicians.

M: Motivation through early demonstration of benefit or areas for improvement.

O: Establish a track record of success.

OR

O2: Monitoring seens as an improvement process and a performance management process

CMO: Early demonstration of direct benefits articulated in a way that is meaningful to clinicians generates motivation that becomes reinforced through supportive feedbacks loops resulting in continued engagement and commitment to the project.

IPT 6 – Information Flow Barriers

C1: Information is not communicated to all stakeholders within the system in a timely and regular manner, particularly for isolated rural clinicians.

OR

C2: Measure not in service level agreements

M1: Feedback is not received in a timely manner that allows clinicians to change practice.

OR

M2: Executives ignore or unaware of measures

O: Habituation and limited diligence and fidelity to the initiative aims.

CMO: Clinicians are unable to respond to early findings or feedback from peers when it is not communicated to them, leading to habituation and limited diligence to continued refinement and scale up of initiative.

LEADERSHIP

Leadership is needed at all levels of large system change initiatives, providing clear direction, supervision, coordination of effort and support.

IPT 1 – Initiative Visibility

C: Local leaders actively engage key stakeholders through various forums, which is reinforced by program sponsors at a system level.

M: Raising the profile and visibility of the initiative: Elevated profile and visibility of the initiative locally by informing and involving people from different departments.

O: Keeps the initiative on the agenda and ensures consistency across the LHD

CMO: When local leaders engage key stakeholders through various forums, with reinforcement of the program by sponsors at a system level (MoH and ACI), this raises the profile and visibility of the initiative across departments, which in turn results in keeping the initiative on the agenda and ensuring consistency across the district.

IPT 2 – Brokerage Failure

C: Initiatives are siloed between inpatient and community services, and not integrated into broader organisational priorities.

M: Brokerage failure: Lack of formal broker with local authority to build long term structures to transform initiative into routine work.

O1: Siloed project: Communication and cooperation are constrained between local departments within organisation, without active exec sponsorship.

OR

O2: Burnout due to implementation discretion - variation in how initiatives are performed in different organisations, creating inefficiencies, and reducing the long-term scaling of benefits.

CMO: The lack of time invested by executives means there is no local authority to build long term structures to transform initiative into routine work, leading to a siloed project without facilitated communication between departments. This can lead to unwarranted implementation variation, inefficiencies, and burnout.

IPT 3 – Leveraging Influence

C: Respected**,** credible, consistent, clear and strong local leadership, supported by adequate funding.

M: Leveraging formal, informal and pre-existing influence: leaders work together by leveraging pre-existing personal resources and network ties, formal and informal authority.

O: Stable momentum for the initiative and trust in the leader driving it.

CMO: Respected**,** credible, consistent, clear, and strong local leadership which leverages pre-existing personal resources and network ties, formal and informal authority leads to a stable momentum for the initiative and trust in the leader driving it in spite of stops and starts in funding.

IPT 4 – Implementation to Institutional Transition

C: Inconsistent, absent or unclear leadership (turnover of leadership)

M: Transition from implementation to institutionalisation failure due to reset or absence of priorities

O: Initiative is discontinuous and unstable, lacking clear and consistent direction.

CMO: Sites that have inconsistent, absent or unclear leadership fail to transition from implementation to routine practice due to reset or lack of priorities, leading to a discontinuous and unstable initiative without clear and consistent direction.

IPT 5 – Collective Action

C: Deferring to local clinical champions who are influential and respected by other clinicians.

M: Culture change towards collective ownership of the clinical model of care / initiative.

O: Distributed leadership cultivated throughout the organisation enabling project momentum.

CMO: Local clinical champions who are respected by other clinicians use social influence to foster culture change towards collective ownership of the model of care / initiative leading to distributed leadership being cultivated throughout the organisation enabling project momentum.

IPT 6 – Disenfranchised Leadership

C: Characteristics of leader clash with other clinicians and promote undermining of initiative.

M: Feeling disenfranchised and disillusioned: Frustration towards the clinical lead by those involved in the initiative and hitting roadblocks.

O: Lack of growth and scaling, turnover of clinicians, and potential for chaos.

CMO: When the behaviour of a leader clashes with other clinicians or undermines the initiative and sets up roadblocks, frustration and disillusionment towards the clinical lead develops leading to lack of growth and scaling, and potential for inconsistent, poor or missed care.

RESOURCE PROVISION

Resources (e.g., extra staff for increased workload, to backfill staff involved with capability development activities, additional equipment) are needed to ensure the feasibility of change and to gain clinical staff buy-in.

IPT 1 – External Accountability

C: Formal resource provision agreements (e.g. service-level agreements for LHDs and performance-level agreement for Pillars).

M: external, top down pressure to be accountable for ensuring those resources are used for funded deliverables

O: Resources used to drive initiative priorities, rather than being absorbed into organisation budget.

OR

C: Advanced notice of formal resource provision agreements (e.g., service-level agreements for LHDs and performance-level agreement for Pillars) in the presence of a proactive QI focussed clinical team

M: provides security that their improvement work is being supported and can proceed

O: better outcomes more rapidly

CMO: Formal resource provision agreements exert external, top down pressure to be accountable for the funding provided, leading to use of resources to drive initiative priorities, rather than being absorbed into organisation budget.

IPT 2 – Short-Term Focus

C: Mismatch between funding and need: temporary funding, overly prescriptive model of care/patient cohort/outpatient vs inpatient, workforce challenges (e.g., retention and turnover in rural sites).

M: Difficulties in suitably staffing initiative.

O: Clinicians find a way to run the initiative, but changes are unsustainable rather than becoming business as usual.

CMO: Mismatch between funding and need, especially uncertainty of future funding, or inadequate initial funding, leads to difficulties in suitably staffing the initiative (issues with recruitment, or local sites investing in short term solutions) that fails to lead to sustainable change, resulting in a lack of continuity.

IPT 3 – Inconsistency of Resource Allocation

C1: Program tied to an individual's tenure (e.g., secretary's priority).

OR

C2: Inconsistency in funding decisions by LHD and initiative.
M: Sustainability of initiative questioned, and conflict between program goals and aims of the models of care.

O: Resources spent unsuitably or absorbed into organisation bottom line, preventing coordination and continuity of the model of care.

CMO: If there is uncertainty in the program's longevity, or uncertainty in funding decisions, sustainability of the initiative is questioned. This leads to **r**esources being spent unsuitably or absorbed into the organisation's bottom line, preventing coordination and continuity of the initiative.

IPT 4 – Resource Investment in Local Capacity

C: Organisations that strategically invest in long-term implementation infrastructure (i.e. permanent structures or staff recruitment/retention, and training to embed initiatives within business as usual).

M: Minimum resource threshold reached, enabling stronger clinical engagement in the design and rollout of the initiative.

O: Capacity developed within organisation to hit the ground running and ensure continued scaling/sustainability of the program.

CMO: Organisations that strategically invested in long-term implementation infrastructure to reach the minimum resource threshold, enabled stronger clinical engagement in the design and rollout of the initiative. This meant that capacity was developed within the organisation to hit the ground running and ensure continued scaling/sustainability of the program.

TIGHT LOOSE TIGHT

An approach in which "tight" well-defined targets and expected outcomes are defined together with responsibilities and accountabilities, but with room for local adaptation ("loose") to achieve these results.

IPT 1 – Striking the Balance

C: Minimum standard of clinical care clearly defined but discretion between different options to deliver the initiative provided, depending on operational constraints (e.g. rural workforce challenges), external perturbations to the system (e.g. COVID), or changes to infrastructure over time.
M: Agents in the system work out the non-negotiables and what local improvement needs are.
O: Balance is struck between fidelity and adaptation, allowing local sites to adjust organisational models to deliver initiatives with local skill mix, resourcing and structures, allowing them to run with those that "fly" and drop those that don't.

CMO: Minimum standard of clinical care clearly defined where agents in the system work out the non-negotiables and what local improvement needs are relevant to their context. Balance is struck between fidelity and adaptation, allowing local sites to adjust organisational models to deliver initiatives with local skill mix, resourcing, and structures, allowing them to run with those that "fly" and drop those that don't.

IPT 2 – Unnecessary Variation

C: For initiatives that are implemented without consistent enabling infrastructure across sites or pre-existing structured organisational models for delivering model/standard of clinical care.
M: Organisations may introduce unwarranted variation to match perceived local needs and local resourcing.
O: Variation in local hospital engagement with implementation support agencies that can reduce fidelity, scalability and sustainability because of the emergence of many path dependent forms of adopting the initiatives.

CMO: For initiatives that are implemented without consistent enabling infrastructure across sites or that did not have a clearly defined Model of Care, variation in local hospital commitment and/or engagement with implementation support agencies leads to organisations introducing unwarranted variation to match perceived local needs and local resourcing because of the emergence of many path-dependent forms of adopting the initiatives.

IPT 3 – Less Discretion

C1: Innovation has potential for universal application and is appropriate for context.
C2: Meso-level structures for program support and coordination matured and tailored locally: typically rural sites establishing initiatives from scratch require more support and metro sites that were scaling existing initiatives could more independently pursue their own goals.

M1: Meso-level implementation support agency legitimises the adoption of behaviours and structures of other successful organisations, especially for rural sites establishing initiatives from scratch.
M2: Metro sites scaling existing initiatives leverage existing research and development or transformation teams.
O: Establishment of processes and resources for initiatives: Initiatives are adopted with fidelity.

CMO: Meso-level structures for program support and coordination that have matured and tailored locally: e.g., for rural sites establishing initiatives from scratch and requiring more support or metro sites that were scaling existing initiatives who were more independently pursuing their own goals, the meso-level implementation support agencies legitimise the adoption of behaviours and structures of other successful organisations, resulting in initiatives being adopted with fidelity.

IPT 4 – Span of Control Failure

C: Lack of clarity re. executive agenda, model of care, implementation approaches, pathway to benefits, and role delineation between stakeholders.
M: Span of control failure: Decision makers are unable to anticipate or obtain the necessary knowledge, information and relationships to respond to local conditions.
O: Local opportunities for innovation are not taken advantage of.

CMO: Lack of clarity around executive agenda, model of care, implementation approaches, pathway to benefits, and role delineation between stakeholders, means decision makers are unable to anticipate or obtain the necessary knowledge, information, and relationships to respond to local conditions. This results in missed local opportunities for innovation.

1. Gauld R, Horwitt J, Williams S, Cohen AB. What Strategies Do US Hospitals Employ to Reduce Unwarranted Clinical Practice Variations? American Journal of Medical Quality. 2011;26(2):120-6.
2. Weaver DJ, Sorrells-Jones J. The business case as a strategic tool for change. J Nurs Adm. 2007;37(9):414-9.
3. O'Connell J, Gardner G, Coyer F. Beyond competencies: using a capability framework in developing practice standards for advanced practice nursing. Journal of advanced nursing. 2014;70(12):2728-35.
4. Fraser SW, Greenhalgh T. Coping with complexity: educating for capability. Bmj. 2001;323(7316):799-803.
5. Cairns L, Stephenson J. Capable workplace learning: Brill; 2009.
